# Simple and Rapid Molecular Techniques for Identification of Amylose Levels in Rice Varieties

**DOI:** 10.3390/ijms13056156

**Published:** 2012-05-18

**Authors:** Acga Cheng, Ismanizan Ismail, Mohamad Osman, Habibuddin Hashim

**Affiliations:** 1 School of Biosciences and Biotechnology, Faculty of Science and Technology, Universiti Kebangsaan Malaysia, Bangi 43600, Selangor Darul Ehsan, Malaysia; E-Mails: acgacheng@yahoo.com (A.C.); maniz@ukm.my (I.I.); 2 Kulliyyah of Science, International Islamic University Malaysia, Jalan Istana, Bandar Indera Mahkota, Kuantan 25200, Pahang Darul Makmur, Malaysia; 3 Malaysian Agricultural Research and Development Institute (MARDI), Jalan Paya Keladi/Pinang Tunggal, Pejabat Pos Kepala Batas, Kepala Batas 13200, Pulau Pinang, Malaysia; E-Mail: habib@mardi.gov.my

**Keywords:** amylose content, MetaPhor agarose gel electrophoresis, microsatellite, rice, single-nucleotide polymorphism, *Waxy* gene

## Abstract

The polymorphisms of *Waxy* (*Wx*) microsatellite and G-T single-nucleotide polymorphism (SNP) in the *Wx* gene region were analyzed using simplified techniques in fifteen rice varieties. A rapid and reliable electrophoresis method, MetaPhor agarose gel electrophoresis (MAGE), was effectively employed as an alternative to polyacrylamide gel electrophoresis (PAGE) for separating *Wx* microsatellite alleles. The amplified products containing the *Wx* microsatellite ranged from 100 to 130 bp in length. Five *Wx* microsatellite alleles, namely (CT)_10_, (CT)_11_, (CT)_16_, (CT)_17_, and (CT)_18_ were identified. Of these, (CT)_11_ and (CT)_17_ were the predominant classes among the tested varieties. All varieties with an apparent amylose content higher than 24% were associated with the shorter repeat alleles; (CT)_10_ and (CT)_11_, while varieties with 24% or less amylose were associated with the longer repeat alleles. All varieties with intermediate and high amylose content had the sequence AGGTATA at the 5′-leader intron splice site, while varieties with low amylose content had the sequence AGTTATA. The G-T polymorphism was further verified by the PCR-*Acc*I cleaved amplified polymorphic sequence (CAPS) method, in which only genotypes containing the AGGTATA sequence were cleaved by *Acc*I. Hence, varieties with desirable amylose levels can be developed rapidly using the *Wx* microsatellite and G-T SNP, along with MAGE.

## 1. Introduction

Rice researchers are currently focused on enhancing rice quality as well as improving yield. In Malaysia, 33 modern varieties of rice have been released for commercial planting, including waxy, non-waxy, aromatic and elongating varieties [1]. Nevertheless, the most important components of rice quality are related to its cooking and eating qualities. Amylose content is considered to be the key determinant of the processing, cooking and eating characteristics of rice (*Oryza sativa* L.), and it correlates directly with the volume expansion, water absorption and ultimate firmness of cooked rice [2]. Generally, the amylose content of milled rice is categorized into five classes: waxy (0–2%), very low amylose (3–9%), low amylose (10–19%), intermediate amylose (20–24%) and high amylose (above 24%). Low amylose content is associated with cohesive, tender and glossy cooked rice. In contrast, high amylose content is associated with dry, firm, fluffy and well separated grains of cooked rice [3]. Lower amylose rice is preferable to higher amylose rice (above 20%) because it does not become hard and dry when cooked.

Previous genetic studies have found that amylose content is governed mainly by an allelic series of genes at one locus and by one or several modifier genes with minor effects [4]. However, its inheritance pattern is complex due to cytoplasmic effects, epistasis and the triploid nature of the endosperm [5], as well as environmental effects [6]. Environmental effects were found to have caused variations of up to 6% in amylose content for a given cultivar [7]. Higher levels of amylose are reportedly controlled by either partial or complete dominance. Thus, heterozygotes cannot be identified using phenotypic measurements of amylose content, and this often leads to inefficiency in traditional rice breeding [5,8]. In the study conducted by Chen *et al*. [9], higher air temperature during grain development was found to be associated with a decrease in apparent amylose content of low and intermediate amylose content rice, but with an increase in apparent amylose content of high amylose content rice.

Over the past several decades, various methods have been reported for the determination of amylose content, including iodine binding, near infrared spectroscopy, size-exclusion chromatography and most recently, asymmetric flow field flow fractionation. However, none of these methods have been validated for routine use, and different values of amylose content have been reported using different methods for the same varieties [10]. The utilization of microsatellites could overcome the shortcomings of amylose content measurements due to their co-dominant nature and their ability to reveal high levels of allelic diversity. Furthermore, microsatellites are easily assayed by polymerase chain reaction (PCR) and are amenable to marker-assisted selection (MAS).

Amylose synthesis in the rice kernel is catalyzed by the granule-bound starch synthase (GBSS) encoded by the *Wx* gene on chromosome 6. There are two functional alleles present in the *Wx* locus, namely *wx^a^* and *wx^b^*, which are found mainly in the indica and japonica varieties, respectively. These alleles produce different levels of *Wx* protein in the endosperm, and consequently cause variation in amylose content. The *wx^a^* allele synthesizes higher contents of GBSS and thus the grains have higher apparent amylose content than those expressing the *wx^b^* allele [11]. Previous studies have identified several nucleotide polymorphisms that are associated with the *Wx* gene, including a polymorphic microsatellite (CT)*_n_* and a G–T SNP located at the 5′-leader intron splice site [12,13]. Another two SNPs are present in exon 6 [A-C SNP] and exon 10 [C-T SNP], which exhibit a high correlation of amylose level with *Wx* microsatellite and G-T SNPs [14,15].

The levels of waxy protein and amylose content were observed to be correlated with the excision of the leader intron of the *Wx* transcripts. High amylose varieties contain high amounts of completely processed GBSS mRNA, while lower amylose varieties contain varying ratios of both completely processed GBSS and partially processed transcripts containing the leader intron [12,13]. The G to T mutation at the 5′-leader intron splice site was reported to be responsible for the characteristics of the *wx^b^* allele; having low level of mature *Wx* transcript, GBSS and apparent amylose content [12,15,16]. Ayres *et al.* confirmed the observation that this single-base change could interfere with mRNA processing in 89 US non-waxy varieties; all 30 low amylose varieties tested had the sequence AGTTATA while 59 intermediate and high amylose varieties had AGGTATA. They reported that the G-T mutation could explain 79.7% of the variation in the amylose content of the non-waxy varieties. Previous study has reported that the AGGTATA and AGTTATA sequences at the 5′-leader intron splice site coincided with the presence of the *wx^a^* allele and *wx^b^* allele, respectively [16]. Various studies have shown similar results in which low amylose varieties had the AGTTATA sequence, while the intermediate and high amylose varieties had the AGGTATA sequence [12,17–21].

Nevertheless, the G-T SNP alone is not sufficient to explain all of the observed genetic variations in amylose content among rice varieties. As an additional explanation on the variation in amylose content, Larkin and Park reported a sequence change in exon 6 of the *Wx* gene, comprised of an A-C polymorphism, which resulted in an amino acid substitution and this was associated with intermediate apparent amylose content rice. This A-C SNP was genotyped together with the G-T SNP in the study conducted by Chen *et al*. using 171 rice accessions, together explaining 86.7% of the variation in apparent amylose content, which characterized rice into low, intermediate and high apparent amylose content classes. Another identified SNP in the *Wx* gene was found to be located in exon 10 and comprised of a C-T polymorphism. The C to T substitution was identified in high apparent amylose rice [14]. All these SNPs could be used as tools to discriminate the different classes of amylose and thereby useful in breeding programs to develop rice varieties with desirable amylose levels.

The present study sought to analyze the *Wx* microsatellite and G-T SNPs at the 5′-leader intron splice site in 15 waxy and non-waxy rice varieties with a wide range of amylose contents. This is an essential first step towards the development of high quality local rice varieties with intermediate amylose contents using molecular approaches. Further, this study aimed to optimize a simple and reliable electrophoresis method, MAGE, which has not been extensively used for the detection of microsatellite alleles, particularly the *Wx* alleles.

## 2. Results

### 2.1. Microsatellite Analysis

**Figure 1:**
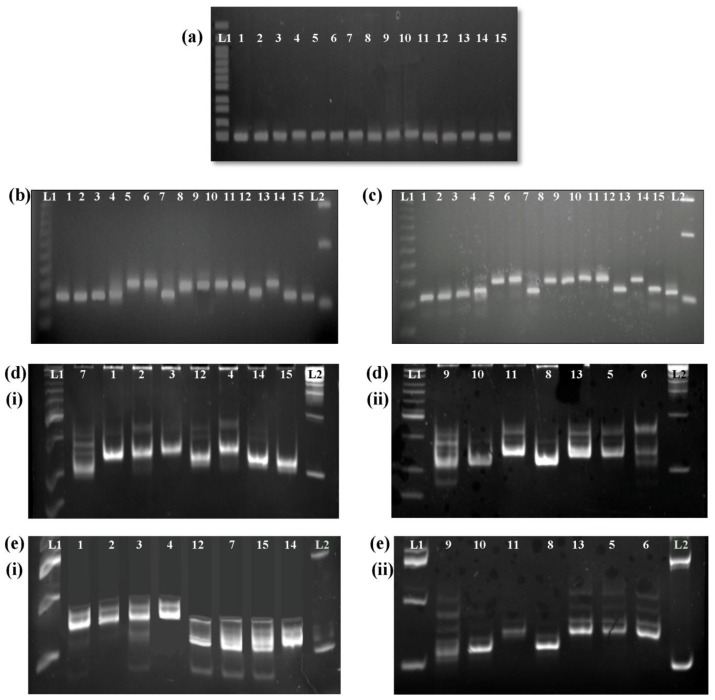
Amplified *Waxy* (*Wx*) microsatellite sequences, separated using (**a**) 1% agarose gel electrophoresis at 100 V for 1 h; (**b**) 4% MetaPhor gel electrophoresis at 80 V for 2 h; (**c**) 4% MetaPhor gel electrophoresis at 80 V for 4 h. Results for PAGE are shown in Figure 1 together with standard agarose and MAGE; (**d**) (**i**) and (**ii**) 8% polyacrylamide gel electrophoresis at 80 V for 2 h; (**e**) (**i**) and (**ii**) 8% polyacrylamide gel electrophoresis at 80 V for 4 h. L1, 100 bp ladder; L2, 25 bp ladder; 1, MR84; 2, Mahsuri Mutant; 3, MRQ74; 4, MR219; 5, MR232; 6, MR263; 7, Basmati 370; 8, Y1304; 9, Khaw Dawk Mali; 10, Pulut Siding; 11, Pulut Hitam; 12, Ria; 13, Mahsuri; 14, Setanjung; 15, MR167.

We screened 15 rice varieties with different amylose contents using primers flanking the *Wx* microsatellite (Table 1). Figure 1 shows the separation of the PCR products as amplified by the *Wx* microsatellite after electrophoresis for (a) 1 h at 100 V on a 1% agarose gel; (b) 2 h at 80 V on a 4% MetaPhor gel; (c) 4 h at 80 V on a 4% MetaPhor gel, PAGE method has been included in the microsatellite analysis; (d) (i) and (ii) 2 h at 80 V on 8% polyacrylamide gels; and (e) (i) and (ii) 4 h at 80 V on 8% polyacrylamide gels, respectively. We did not observe polymorphic bands between any of the tested varieties on the standard agarose gel. However, we observed polymorphic bands between the tested varieties on the 4% MetaPhor and 8% polyacrylamide gels. Two hours of electrophoresis barely separated the alleles, but the alleles were separated clearly after 4 h of electrophoresis for both MAGE and PAGE. Thus, the small size differences between the *Wx* microsatellite alleles required longer electrophoresis periods for clear resolution.

The amplified products ranged from 100 to 130 bp in length and contained between 10 and 18 (CT)*_n_* (Table 1). Five classes of *Wx* microsatellite alleles were detected: (CT)_10_, (CT)_11_, (CT)_16_, (CT)_17_, and (CT)_18_. The (CT)_11_ and (CT)_17_ were the predominant classes of (CT)*_n_* repeats in this study. Ten out of the fifteen tested varieties tested contained the (CT)_11_ and (CT)_17_ alleles. All of the varieties with the (CT)_11_ allele had high amylose content (above 24%) and varieties with the (CT)_17_ allele had low and intermediate amylose content (10–19%), with the exception of the waxy rice Pulut Hitam 9 (0.34% amylose). All of the genotypes analyzed were homozygous for the *Wx* microsatellite allele.

### 2.2. Sequence Analysis

**Figure 2:**
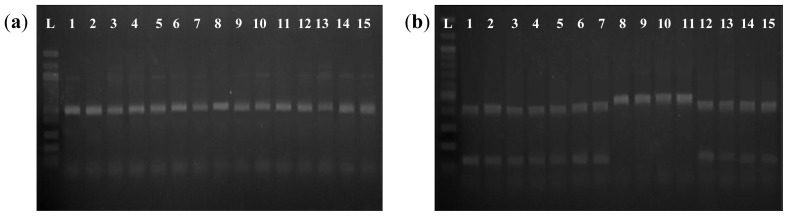
Amplified PCR-*Acc*I cleaved amplified polymorphic sequence (CAPS) products (**a**) before and (**b**) after *Acc*I digestion. L, 100 bp ladder; 1, MR84; 2, Mahsuri Mutant; 3, MRQ74; 4, MR219; 5, MR232; 6, MR263; 7, Basmati 370; 8, Y1304; 9, Khaw Dawk Mali; 10, Pulut Siding; 11, Pulut Hitam; 12, Ria; 13, Mahsuri; 14, Setanjung; 15, MR167

To study the relationship between the *Wx* microsatellite allele and the G-T SNP, all PCR products of the 15 tested varieties were sequenced in the region containing the *Wx* microsatellite and the 5′-leader intron splice site. Interestingly, all varieties with intermediate (20–24%) and high amylose content (above 24%) had the sequence AGGTATA at the splice site, while all waxy (0–2%) and low-amylose content (below 19%) varieties had the sequence AGTTATA. The sequencing results were further validated using a restriction enzyme digestion assay, in which the amplified products were digested with *Acc*I. All amplified products containing the sequence AGGTATA were cleaved by *Acc*I, resulting in two fragments of expected size. In contrast, the genotypes containing AGTTATA were not cleaved and remained as a single fragment (Figure 2). This indicates that the G-T polymorphism can be assayed either through sequencing or PCR-*Acc*I cleaved amplified polymorphic sequence (CAPS) to differentiate waxy and low amylose varieties from intermediate and high amylose varieties.

## 3. Discussion

Previous studies have reported that a polymorphism in the *Wx* microsatellite located 55-bp upstream of the putative 5′-leader intron splice site in the *Wx* gene was responsible for more than 82% of the variation in the amylose content of non-waxy rice [4,14]. This indicates that the *Wx* microsatellite can be used to distinguish most rice varieties with different amylose contents.

PAGE method and its limitations: The most common electrophoresis method employed in previous studies has been PAGE, due to its ability to separate many samples simultaneously with scoring accuracy [4,14,21]. However, PAGE is technically challenging and the process can be cumbersome and time-consuming. Polyacrylamide gels are thin and fragile, and the glass plates used in casting them are difficult to handle. We worked on both 5% (data not shown) and 8% polyacrylamide gels and we found that 5% polyacrylamide gels are rather crumbly and easily broken. Besides, acrylamide is carcinogenic and is known to be a cumulative neurotoxin.

The advantages of MAGE over PAGE: In this study, MAGE was used instead of PAGE due to its ability to produce thicker and more stable gels and the fact that it uses a standard agarose gel electrophoresis system. We found these gels easier to prepare and free from any leakage problems during gel casting. This method was approximately 4 h shorter than the PAGE method employed by Jayamani *et al*. [21], and like PAGE, it is able to separate many samples simultaneously with good scoring accuracy. According to Ochsenreither *et al*. [22], PAGE and MAGE are both currently used for microsatellite analysis and they produce comparable and reproducible results. In our results, however, MAGE gives clearer single bands for the *Wx* microsatellites with no shadow bands as compared with PAGE.

A total of 16 *Wx* microsatellite alleles, ranging in length from (CT)_4_ to (CT)_22_, have been found to be correlated with amylose content [4,14,21,23,24]. In the present study, fifteen varieties that are commonly used in local breeding programs were characterized using *Wx* microsatellite and G-T SNP analysis. Five microsatellite alleles of the *Wx* gene were identified, namely (CT)_10_, (CT)_11_, (CT)_16_, (CT)_17_, and (CT)_18_. All of the selected varieties with high amylose content (above 24%) were associated with shorter repeat alleles, namely (CT)_10_ and (CT)_11_, while longer repeat alleles, including (CT)_16_, (CT)_17_ and (CT)_18_, were detected in the selected varieties with low and intermediate amylose contents (below 24%).

Of the five microsatellite alleles detected, (CT)_11_ and (CT)_17_ were found in the most tested varieties. Studies conducted by Ayres *et al.* and Bergman *et al.* found that varieties with the (CT)_11_ allele had an amylose content of greater than 23%, consistent with the results obtained in this study, in which all varieties with the (CT)_11_ allele were high amylose varieties with an apparent amylose content greater than 24%. According to Ayres *et al.*, the (CT)_17_ allele is present in varieties with divergent amylose contents. In this study, the (CT)_17_ allele was present in a waxy variety, Pulut Hitam 9, which has an amylose content of 0.34%, but it was also found in three varieties with apparent amylose contents of 14 to 19.6%, including MR263 and both the internationally accepted high quality varieties (Khaw Dawk Mali and Basmati 370). Our results were similar to those of previous studies. The (CT)_17_ allele was reported to be prominent in the Thai rice germplasm; where 36 out of 68 Thai rice strains tested contained this allele, including Khaw Dawk Mali 105 [25]. The (CT)_17_ allele was also present in all eight Basmati accessions tested by Jayamani *et al.* We also found that the *Wx* microsatellites could not always be used to distinguish the waxy varieties, as concluded by Ayres *et al.* The two waxy varieties tested, Pulut Siding and Pulut Hitam 9, had different alleles, (CT)_16_ and (CT)_17_, respectively. However, the waxy varieties were found to have larger repeat alleles, (CT)*_n_*, *n* > 15, as did other low amylose varieties.

To study the correlation between the *Wx* microsatellite allele and the G-T SNP, we sequenced the region containing the microsatellite from all selected varieties representing all five detected microsatellite classes. The present results were consistent with those of previous studies, which found that all intermediate and high amylose varieties had the sequence AGGTATA, while waxy and low-amylose varieties had the sequence AGTTATA [4,15,17,19,21,25]. We found that all varieties with the (CT)_10_, (CT)_11_ and (CT)_18_ alleles had the sequence AGGTATA, whereas all varieties with (CT)_16_ had the sequence AGTTATA. (CT)_17_ was the only allele that could be associated with either the AGGTATA or AGTTATA sequences. The presence of both sequences in the (CT)_17_ was also observed by Jayamani *et al.* [21] and Chen *et al.* [26].

The sequencing results of the G-T SNP at the 5′-leader intron splice site were further confirmed using the PCR-*Acc*I CAPS method. In all cases, amplified fragments that contained the sequence AGGTATA in the splice site were cleaved by *Acc*I, resulting in two fragments of the expected size on a 1.5% agarose gel, whereas amplified fragments containing the sequence AGTTATA were not cleaved by *Acc*I and remained as a single fragment (Figure 2). We noted that the PCR-*Acc*I CAPS method is effective in the detection of the G-T SNP and took less time than the sequencing reactions to discriminate waxy and low amylose varieties from varieties intermediate and high amylose varieties. G-T SNP analysis has been applied in breeding programs to produce indica varieties with lower amylose contents [6]. Ayres *et al.* reported that the G-T SNP could explain approximately 80% of the variation in amylose content in non-waxy rice varieties. In this study, the G-T SNP could distinguish varieties with 18% or less amylose from those with higher than 18% amylose with 100% accuracy. However, this G-T SNP could not be used to discriminate between intermediate and high amylose varieties.

Based on the present study, the combination of *Wx* microsatellite allele and G-T SNP identification could be used to differentiate high amylose (above 24%), intermediate amylose (20–24%) and low amylose or waxy varieties (below 19%). The *Wx* microsatellite allele could be used to discriminate high amylose varieties from intermediate and low amylose varieties. All of the selected varieties with high amylose contents had shorter repeat alleles, *i.e.*, (CT)*_n_*; *n* = 10 and 11, whereas the selected varieties with 24% or less amylose contents had longer repeat alleles, *i.e.*, (CT)*_n_*; *n* = 16, 17 and 18. Meanwhile, the G-T SNP could be used to discriminate intermediate and high amylose varieties from waxy and low amylose varieties. Hence, the *Wx* microsatellite and G-T SNP analysis can be used together as tools to develop rice varieties with the desired amylose levels through breeding work supported by MAS.

## 4. Experimental Section

Fifteen varieties were used in the study, including two internationally accepted quality rice varieties: Basmati 370 and Khaw Dawk Mali. Seeds were obtained from the Malaysian Agricultural Research and Development Institute (MARDI). The selected varieties were classified as waxy (0–2%); low amylose (below 19%); intermediate amylose (20–24%) and high amylose (above 24%) based on the data provided by MARDI (Table 1). Apparent amylose content was estimated using the method described by Juliano (1971).The apparent amylose content (%) was estimated according to Juliano [27]. The seeds were sown and grown in the greenhouse and leaves of 2- to 4-week-old seedlings were harvested for DNA isolation. Genomic DNA was extracted using the cetyltrimethyl ammonium bromide (CTAB) method described by Murray and Thompson [28] with minor modifications. The quality of DNA samples was examined using agarose gel electrophoresis.

For the *Wx* microsatellite assay, PCR was performed in 25 μL reaction mixtures, containing the respective 0.25 μg of template DNA, 1× Green GoTaq flexi buffer, 1.5 mM MgCl_2_, 0.2 mM deoxynucleotides, 0.5 μM oligo 484 (forward primer: 5′-CTTTGTCTATCTCAAGACAC-3′), 0.5 μM oligo 485 (reverse primer: 5′-TTGCAGATGTTCTTCCTGATG-3′) [4] and 2.5 units of GoTaq DNA polymerase (Promega, Madison, WI). The targeted fragments were amplified using a Mastercycler Gradient (Eppendorf, Hamburg) with the following steps: Denaturation at 95 °C for 4 min, followed by 35 cycles of 94 °C for 45 s, 55 °C for 30 s and 72 °C for 60 s, and 5 min at 72 °C for the final extension. After PCR, 5 μL of the PCR products was evaluated on a standard 1% agarose gel to confirm successful amplification. The remaining 20 μL was separated on 4% MetaPhor agarose gels and 8% polyacrylamide gels.

For the 4% MetaPhor gel preparation, 2% MetaPhor agarose (Lonza, Walkersville, MD, USA) and 2% standard agarose (First Base, Singapore, SG) were slowly sprinkled into 1× TAE buffer with rapid stirring. The agarose was soaked in the buffer for 15 min before heating to prevent the solution from foaming during heating. The solution was heated in a microwave at medium power for 2 min before reheating at high power for 1 min. Once the molten gel was cast and solidified, it was placed at 4 °C for 20 min prior to use for optimal resolution. The *Wx* microsatellite PCR products were electrophoresed for 2 or 4 h at 80 V on ice and stained with an ethidium bromide solution for 10 min followed by 20 min of destaining with water. The gels were observed under UV transillumination (Alpha-Imager). The size of the amplification products was estimated by comparisons to 25-bp and 100-bp DNA ladders (New England Biolabs, Ipswich, MA, USA).

PAGE protocol: For the 8% polyacrylamide gel preparation, 2.4 mL of Acrylamide/Bis 19:1 40% (Ambion, Woodward Austin, TX) were added to 2.4 mL of 5× TAE buffer and 7.2 mL of distilled water to give a total volume of 12 mL. Two hundred microliters of 10% ammonium persulfate (Sigma, Saint Louis, MO, USA) and 10 μL of N,N,N′,N′-tetramethylethylenediamine (Sigma, Saint Louis, MO, USA) were then added to the mixture to complete the gel before the acrylamide polymerized. Once the molten gel was cast and solidified, it was placed at 4 °C until needed prior to use for optimal resolution. The *Wx* microsatellite PCR products were electrophoresed for 2 or 4 h at 80 V and stained with an ethidium bromide solution for 5 min followed by 15 min of destaining with 1× TAE buffer. The gels were observed under UV transillumination (Alpha-Imager). The size of the amplification products was estimated by comparisons to 25-bp and 100-bp DNA ladders (New England Biolabs, Ipswich, MA, USA).

For sequencing analysis, the same PCR reaction conditions were used to obtain a larger amplified fragment from a subset of all tested varieties, using oligo 484 and W2R (reverse primer: 5′-TTTCCA GCCCAACACCTTAC-3′) [4]. The PCR products were electrophoresed on a 1.5% agarose gel and the resulting bands were excised. The amplified products were purified using a QIAquick Gel Extraction Kit (Qiagen, Valencia, CA) according to the manufacturer’s protocol and were sequenced (Macrogen, Rockville, MD). To further confirm the presence or absence of the G-T polymorphism at the 5′-leader intron splice site, 10 μL of each amplified product obtained from PCR-*Acc*I CAPS (forward primer: 5′-ACCATTCCTTCAGTTCTTTG-3′; reverse primer: 5′-ATGATTTAACGAGAGTTGAA-3′) [29] was digested with 2 units of *Acc*I (New England Biolabs, Ipswich, MA, USA) in a total volume of 15 μL for 2 h at 37 °C. The samples were electrophoresed for 1.5 h at 80 V in a standard 1.5% agarose gel.

## 5. Conclusions

In this paper, we demostrated that MAGE is a rapid and reliable method for separating *Wx* microsatellite alleles. In addition, we report that the combination of *Wx* microsatellite and G-T SNP polymorphism analysis could efficiently discriminate between low, intermediate and high amylose varieties. These molecular markers are being used in ongoing research and breeding works for the development of local high quality rice varieties with intermediate amylose contents.

## Figures and Tables

**Table 1:** Apparent amylose content, *Waxy* microsatellite length and G-T single-nucleotide polymorphism of 15 important and commonly used rice varieties in the local breeding programs.

Variety	Apparent amylose content (%)	Amylose class	(CT)*_n_*	G-T
Ria	28.7	High	11	G a
MR84	28	High	10	G a
Setanjung	28	High	11	G a
MRQ74	27	High	11	G a
Mahsuri	26.9	High	10	G a
Mahsuri Mutant	26	High	11	G a
RM167	26	High	11	G a
MR219	21	Intermediate	17	G a
MR232	20	Intermediate	18	G a
Basmati 370	19.6	Intermediate	17	G a
MR263	18	Low	17	G a
Y1304	14	Low	16	T
Khaw Dawk Mali	14	Low	17	T
Pulut Siding	1.4	Waxy	16	T
Pulut Hitam 9	0.34	Waxy	17	T

a The amplified products were digested by *Acc*I restriction enzyme which further confirmed the sequencing results on G-T polymorphism.
